# Digital Literacy Training for Digitalization Officers (“Digi-Managers”) in Outpatient Medical and Psychotherapeutic Care: Conceptualization and Longitudinal Evaluation of a Certificate Course

**DOI:** 10.2196/70843

**Published:** 2025-08-29

**Authors:** Anne Mainz, Timo Neunaber, Paula Cara D'Agnese, Alexander Eid, Tanja Galla, Christoph Ellers, Sven Meister

**Affiliations:** 1 Health Informatics, Faculty of Health, Witten/Herdecke University, Pferdebachstr. 11, Witten, 58448, Germany, 49 230292678627; 2 IT & Digital Health Division, Kassenärztliche Vereinigung Westfalen-Lippe, Dortmund, Germany; 3 Department of Education, Ärztekammer Westfalen-Lippe, Münster, Germany; 4 Department of Healthcare, Fraunhofer Institute for Software and Systems Engineering, Dortmund, Germany

**Keywords:** digital competence, digital literacy, digital maturity, certificate course, training course, longitudinal study, medical assistant, health care professional

## Abstract

**Background:**

Digital tools, services, and information in patient care demand new competencies in outpatient care, and the workforce is faced with the need to deal with digitalization.

**Objective:**

In a targeted certificate course (Certification of Digitalization Officers in Medical Practices and Psychotherapeutic Practices, Digi-Manager), medical assistants are trained to serve as digitalization officers, enabling them to implement the requirements of digitalized health care within their practices.

**Methods:**

As part of an accompanying study, the course is evaluated by the participants, and the change in their digital literacy is recorded. We measured different knowledge, skills, and attitude dimensions at 3 different times—before, during, and after the course—and used ANOVA to examine significant changes.

**Results:**

Digi-Managers started the course with an already high self-assessment of their digital literacy. Skills and knowledge increased significantly in all categories (cognitive, technical, ethical, and health information) from the initial to the final measurement, as did self-confidence in the use of general software and hardware. Positive attitude remained stable over the training period, and the course was rated very positively by participants across all areas.

**Conclusions:**

Training programs on digital topics for health care professionals are necessary, and this certification course is a role model for successful further education through a mixture of theoretical knowledge transfer and practical application. Especially, the use of a digital maturity model and a digital laboratory was a unique and useful feature. Further research needs to go into alternative assessment methods of digital literacy, as the results suggest that self-assessment measures self-efficacy and confidence, rather than pure competence. Nevertheless, the increase in self-assessed competence suggests that the training was successful.

## Introduction

### Background

The use of digital innovations with different tools, services, and information in patient care is associated with new requirements for day-to-day work in outpatient medical care, and the spectrum of them is expanding more and more. Not only physicians but also other medical professionals are faced with the need to deal with digitalization [1]. Telemedicine, eHealth, and mobile health (mHealth) enable access to health information, improve communication, allow personalized care, make remote monitoring possible, and support self-management of patients [2]. Administrative processes are also increasingly supported digitally [1].

The digital transformation in Germany’s health care system has led to a few important digital tools in recent years: the electronic patient record (ePA), electronic sick leave certificates (eAU), electronic prescription (eRezept), the electronic medication plan (eMP), electronic communication systems (KIM and TIM), telehealth applications, and digital health applications (DiGA). Additionally, various legal initiatives to establish more technologies in health care are currently coming into force in Germany [3]. On the side of the outpatient medical practices in Germany, the level of digitization or digital maturity depends heavily on the individual personality, motivation, and competence of the people involved [4-6]. You will find mostly outdated IT systems within the practices [3], and many things still happen in paper form, such as the storage of data or communication with other health care providers or patients [4].

Besides the availability of digital tools, services, and information, individuals need more motivation to use these technologies. Following the self-determination theory [7], the feeling of competence is one of the three pillars to develop motivation. Otherwise, feelings of incompetence regarding the use of health informatics technology tools lead to reluctance in using these tools and are one of the main reasons to avoid them. Digital competence or digital literacy in health care could be defined as the ability to integrate and apply context-appropriate knowledge, skills, and psychosocial factors—such as attitudes, beliefs, values, and motivation—to perform within the health care domain [8]. Especially when health care workers were not given enough time to learn on-the-job or did not have enough support from peers, they were not willing to use new technology [9]. To enable individuals to gain digital literacy in health care, education is needed.

In their focus group study, Mannevaara et al [10] identified knowledge and skill issues regarding IT-related management and IT background knowledge as the main challenges faced in health care. Competencies related to direct patient care, communication, ethics in health IT, project- and change management, digital literacy, information and knowledge management, teaching, and education were essential in today’s health care practice. Especially, competencies in decision-making, information and knowledge management, teaching, training, and data security were highlighted as important by German participants in this study [10]. Hübner et al [11] identified the top 3 core competency areas that need to be addressed for employees in the health care sector that work in direct patient care: (1) communication, (2) documentation, and (3) information and knowledge management in patient care, which coincides with the focus of the Digi-Manager training course.

### Goal of This Study

With this in mind, the project “Certification of Digitalization Officers in Medical Practices and Psychotherapeutic Practices (Digi-Manager),” funded by the Federal Ministry of Health in Germany, launched an educational program for medical assistants to train them as digitalization officers. In Germany, medical assistants make appointments for patients, document treatment procedures for patient files, take care of billing for services rendered, and organize practice procedures. They apply bandages, prepare syringes, or draw blood for laboratory tests. They also inform patients about pre- and posttreatment options, maintain medical instruments, and carry out laboratory work [12].

Digitalization officers, or how they are called in this certification program, “Digi-Managers,” create digitalization strategies for their own practices, can get digitalization projects off the ground, and act as a point of contact for the digitalization of patient care.

To evaluate if the digital literacy of the participants could be increased through participation in the Digi-Manager course, their digital literacy before (t0), during (t1), and after (t2) the course was measured and compared. To measure the whole concept of digital literacy, participants are asked about their knowledge, skills, and attitudes regarding digital health technologies.

### Hypotheses

The transfer of skills and abilities should be verifiable in a successful training program, which is why hypothesis H1 is formulated. A scoping review [13] suggests that discussions, group workshops, self-directed learning materials, and providing practice opportunities, among other things, help to grow digital confidence, which are provided within the Digi-Manager training. This leads to our hypothesis H2. Other studies show that increased usage causes a rise in computer confidence, which also increases positive attitudes toward computers [14], which supports the assumption of hypothesis H3.

H1: Attending the Digi-Manager training course significantly improves the specific knowledge and skills imparted to participants.
H2: Attending the Digi-Manager training course significantly improves participants’ general confidence level for technology usage.
H3: Attending the Digi-Manager training course significantly improves participants’ attitude toward digital (health) applications.

## Methods

### Ethical Considerations

The ethics application was reviewed and approved by the ethics committee of Witten/Herdecke University on April 20, 2023, and no ethical or legal concerns were raised (application/approval no. S-93/2023). The participants received no further compensation for participating. Before each survey, the participants received information about the duration, procedure, and content of the study and had to provide consent. For each question, there was the option to refuse to answer. The responses of the participants were pseudonymized using an identification code, and no identifying information was queried.

### Course Concept

The aim of the Digi-Manager certificate course was to enable medical assistants in outpatient medical practices to derive and implement digitalization strategies and projects for their own practices and become contact persons for the digitalization of patient care. Therefore, they had to acquire skills in the areas of technology, data use, IT security, and data protection.

**Figure 1.**
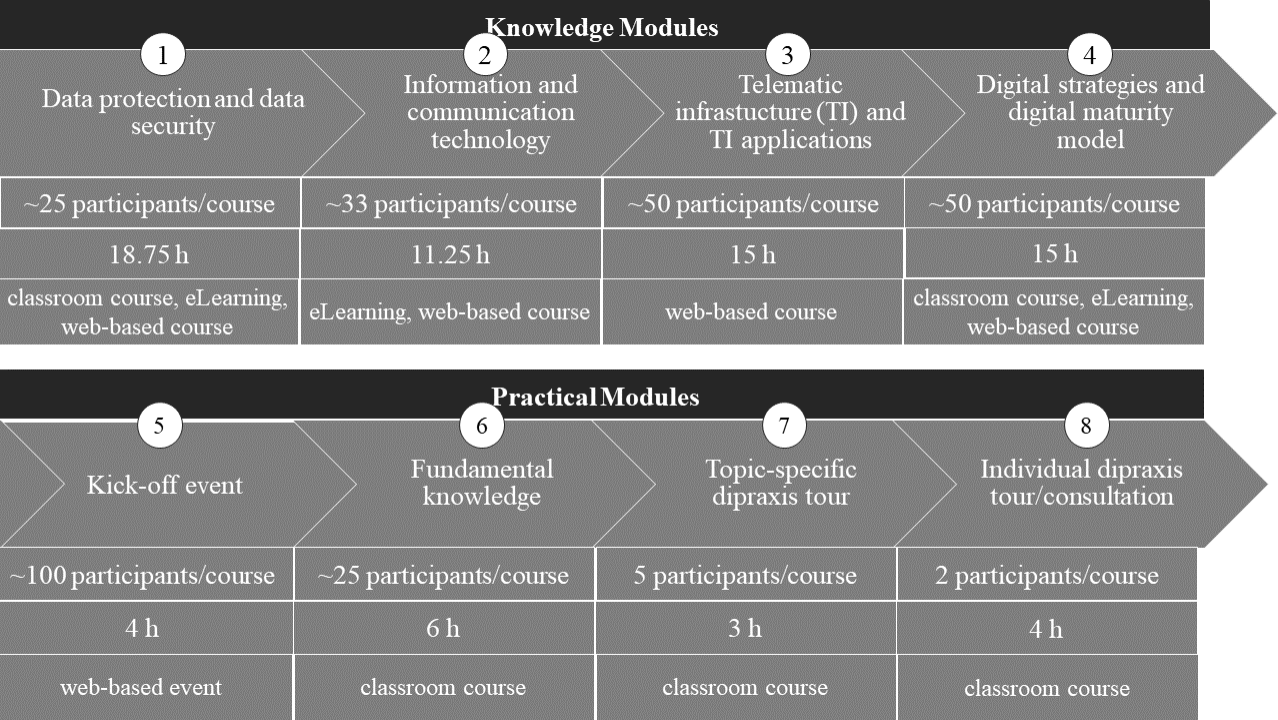
Schedule of the certificate course.

The participants were released from work for a total of 205 hours during the certificate course and received no further incentives for participation. The participants spend 60 hours on the knowledge modules and 17 hours on the practical modules in web-based and on-site classroom courses. Approximately 128 hours were planned additionally for orientation, organization, self-study, exams, and creating a digitalization strategy (Figure 1). Different time slots (2‐4 slots per module) were offered for each course, and participants were allowed to choose them freely. Participation in each module was mandatory to pass the certificate course, and attendance was assessed through an attendance list. The content of the course was developed based on an existing certified course of ÄKWL (Ärztekammer Westfalen-Lippe, a medical association representing 40,000 doctors of the Westphalia-Lippe region) that has been running for many years and was adapted and supplemented by a panel of experts with regard to the requirements of digitalization officers (Multimedia Appendix 1).

The course consisted of so-called knowledge and practical modules. The knowledge modules lasted from May until September 2023 and were held as blended learning courses with e-learning materials and web-based and on-site courses. The e-learning materials and web-based classes were distributed by the learning management platform ILIAS.

The practical modules lasted from October 2023 until May 2024 and consisted of a kick-off event, a fundamental knowledge course, and consultations in smaller groups. The courses took place at the dipraxis, a digital laboratory for testing digital tools and analyzing processes in outpatient care. A major component of the practical modules was the digital maturity model of the KVWL (Kassenärztliche Vereinigung Westfalen-Lippe, an association of statutory health insurance physicians of the Westphalia-Lippe region). The model was developed on the findings of Neunaber and Meister [15] for this project. It measures the digital maturity of the practices on 5 assessment categories: corporate management, infrastructure (IT security, data protection, interoperability, data processing, telematic infrastructure, and data collection), treatment and therapy, patient management, and administration. Course participants classified their own practice by answering 25 items. A digital tool visualized the digitalization status of the practice based on the answers using a radar chart. The model could be used by Digi-Managers to assess the current level of digitalization in the practice and identify potential for improvement of the digital situation. With the results of the digital maturity model and the consulting in the dipraxis, the digital managers developed practice-specific digitalization strategies.

### Recruitment

The respondents for the survey were the participants of the Digi-Manager training course. Of the total of 100 participants, all were asked to take part in the surveys.

### Guideline

The GREET (Guideline for Reporting Evidence-Based Practice Educational Interventions and Teaching) checklist was used to describe the educational intervention in detail [16]. It comprises 17 items to describe why, what, who, how, where, when, how much, and how well an educational intervention took place and what planned and unplanned changes occurred.

### Questionnaires

Within the initial survey (Multimedia Appendix 2), participants were asked about their basic demographic information to describe the study population and identify possible biases: their age, gender identity, duration of employment, and the medical field of the doctor’s office they work at. Various studies indicate that age and gender are associated with different usage behavior and perception of digital tools [17,18], but other studies show that the influence of these variables is often overestimated [19,20]. In order to control these possible effects, demographic information was collected and analyzed.

Digital literacy of the participants was measured using 2 existing questionnaires, which recorded both the skills and knowledge relating to digital (health) systems as well as attitudes toward digital (health) systems. Since digital literacy is understood as the competence to deal with digital tools in the everyday professional life of medical assistants in this study, an instrument was needed that operationalizes different dimensions of competence and refers to the wide range of digital tools in medical practices. Fitting tools in terms of content and quality criteria were identified using the results of a preceding scoping review [21]. The first questionnaire used was the Public Health Informatics Competencies for Primary Health Care (PHIC4PHC) questionnaire [22], with a total of 42 items on a 5-point Likert scale. The items are divided into the following dimensions: *cognitive proficiency* (digital health system knowledge and digital health system skills), *technical proficiency* (general computer skills, office application skills, and network skills), *ethical proficiency* (privacy, security, and legal knowledge), and *health information literacy* (health information access, management, integration, and evaluation).

In addition to knowledge and skills, the questionnaire by Kuek and Hakkennes [23] was used to record the attitude toward the use of digital health care systems via items on (1) confidence in usage behavior, (2) technology acceptance, and (3) acceptance and use of technology.

The first part of the questionnaire required participants to indicate their confidence level for different commonly used hardware and software devices on a 5-point scale from not at all confident to completely confident. The devices and applications in question were computers, office applications, smartphones, tablets, email, and social media. The second and third parts of the questionnaire were based on the TAM (technology acceptance model) and UTAUT (unified theory of acceptance and use of technology) questionnaires, which have been used and validated in various studies. Technology acceptance of health information systems was measured using the dimensions *perceived usefulness* and *perceived ease of use* of the TAM, with 12 items, and supplemented with the dimensions *attitude toward technology*, *social influence*, *facilitating conditions*, and *anxiety* of the UTAUT, with 15 items, all on a 7-point Likert scale.

The system usability scale (SUS) was used as an established, standardized, and quick questionnaire for the assessment of perceived usability of the digital maturity level tool (Brooke, 1996, quoted from [24]). The 10 items were measured on a 5-point Likert scale.

### Procedure

Digital literacy was assessed in the form of a longitudinal study parallel to the certificate course. The measurement points were based on the participants’ progress in the course: before completing the first knowledge module (t0), after completing all 4 knowledge modules (t1), and after completing the practical modules (t2). The links to the questionnaires were distributed via the learning management platform ILIAS, which was used to provide communication and content for the Digi-Manager training. This made it easier for participants to access the surveys, and the integrated reminder and activation functions ensured that participation in the surveys was not forgotten. In addition, the completion of the evaluation surveys could be used as a condition for further progress within the learning material in ILIAS. As no further learning content was planned after the final survey, reminder emails were sent to the participants for this purpose.

Before starting every survey, the participants were informed about the content of the study, data processing, and data protection and were asked for their written consent to participate. To statistically evaluate the change in digital literacy without violating anonymity, participants were asked to provide each survey with their individual identification code. This code was a 6-digit character string made up of time-stable personal characteristics.

The first questionnaire for t0 collected basic demographic information and measured the participants’ existing digital skills. The second questionnaire for t1 was intended to assess the digital literacy of the participants again and additionally asked about the evaluation of the first part of the training. At this second measurement point, the Digi-Managers also had the opportunity to evaluate the certificate course in terms of support and design (participant support, technical moderation, quality of scripts, and atmosphere), content (topicality of content, content structure, selection of speakers and authors, discussion/interaction, practical relevance, and personal goal achievement), planning and organization (program announcement, selection of dates, and time frame), and the web conferencing system (technical functionality, user friendliness of the screen, sound quality, and image quality). Participants were able to rate the various aspects of these dimensions on a scale of 1 to 6, with 1 as the best and 6 as the worst rating option. In addition, there were free-text questions in which the participants were asked what they perceived as particularly positive or particularly negative about the training. In the final questionnaire for t2, digital literacy was assessed one last time, the second part of the training was evaluated, and the applicability and comprehensibility of the digital maturity level tool were recorded.

### Data Analysis

The datasets of the different surveys were matched through the 6-digit individual identification code. Since some data records did not have fully matching codes, data records were also assigned that were at most 2 characters different or in a reversed order.

The collected data were analyzed with the IBM SPSS Statistics 28 analysis software. To analyze the data descriptively, the frequency, mean values, and standard deviations were reported. Normal distribution as a prerequisite was tested by the Shapiro-Wilk test because it is more powerful than the Kolmogorov-Smirnov test [25], and sphericity was tested with the Mauchly test. The Levene test was used to test the homogeneity of variances.

Correlations were tested through Pearson *r* and rated with the classification from Cohen [26], with |*r*|=0.1 as weak correlation, |*r*|=0.3 as moderate correlation, and |*r*|=0.5 as strong correlation. ANOVA with repeated measures was used to test if the mean values of digital literacy at different measurement points differ significantly. Post hoc tests were used to determine between which measurement times significant differences exist. For evaluation of effect sizes, the classification according to Cohen [26] was chosen with 0.01 as a weak effect, 0.06 as a moderate effect, and 0.14 as a large/strong effect. As η² systematically overestimates the effect size, ω² and ε² are also calculated, as these have lower bias [27]. Between-subject effects such as age, gender, and education level were also tested to gain insights into their additional effect.

The SUS score was calculated as the sum of item scores with the negative worded items inverted and multiplied by 2.5, resulting in a value between 0 and 100 [24].

The free-text answers were formed into inductive categories, and only the top 3 of particularly positive or particularly negative aspects were reported. All other named categories could be seen in Multimedia Appendix 3.

## Results

### Digi-Manager Characteristics

A total of 100 participants started the Digi-Manager training course. The participants were aged between 20 and 61 years, with an average age of 37.4 (SD 11.3) years, and the vast majority identified as female (95/100, 95%). Only 4 (4%) identified as male, and 1 (1%) did not provide any information on their gender identity. The participants worked at doctors’ offices with different primary medical fields, as shown in Table 1.

**Table 1.** Participants’ demographic data (N=100).

Characteristics	Participants (N=100)
Age (years), mean (SD)	37.4 (11.3)
Gender, n (%)	
Woman	95 (95)
Man	4 (4)
Prefer not to say	1 (1)
Primary medical field of doctor’s office, n (%)	
General practice	50 (50)
Gynecology	9 (9)
Dermatology	5 (5)
Orthopedics	5 (5)
Psychiatry or psychotherapeutic practice	4 (4)
Pediatrics	3 (3)
Internal medicine	3 (3)
Neurology	3 (3)
Urology	2 (2)
Ophthalmology	2 (2)
Gastroenterology	2 (2)
Child and adolescent psychiatry	2 (2)
Oral and maxillofacial surgery	2 (2)
Otorhinolaryngology	1 (1)
Pneumology	1 (1)
Nuclear medicine	1 (1)
Radiology	1 (1)
Reproductive medicine	1 (1)
Not specified	3 (3)

For the first survey, the participation rate was 100%. In the second survey, 97 out of the 100 Digi-Managers participated. For the third and last survey, only 64 of the 100 answered the entire questionnaire, despite repeated reminders via email and ILIAS.

Gender as a between-subject effect was not further monitored because of the vast majority (95/100, 95%) of female-identifying participants. The variables that showed a significant correlation with age were all 3 measurement points for confidence in usage behavior with a medium negative effect (*r*_t0_=–0.422, *P*<.001; *r*_t1_=–0.423, *P*<.001; *r*_t2_=–0.381, *P*=.005). The other correlation values can be seen in Multimedia Appendix 4.

### Evaluation of the Certificate Course

The course was evaluated separately for the knowledge and the practical modules, but for both, the review was very positive. Most ratings were equal to or above a mean value of 2; only the *selection of dates* for the knowledge modules and the *time frame* for both the knowledge and practical modules were just below this value. All other categories for support and design, content, and web conferencing systems were ranked 1.4‐2 (Table 2).

**Table 2.** Evaluation of the certificate course aspects by the Digi-Managers (scale from 1 (very good) to 6 (very bad)).

Evaluation categories	Knowledge modules, mean (SD)	Practical modules, mean (SD)
Support and design		
Participant support	1.7 (0.9)	1.4 (0.7)
Technical moderation	1.6 (0.7)	—^a^
Quality of scripts	1.9 (0.9)	—
Atmosphere	1.7 (0.8)	1.4 (0.7)
Content		
Topicality of content	1.6 (0.6)	1.4 (0.7)
Content structure	1.8 (0.8)	1.7 (0.8)
Selection of speakers/authors	1.9 (0.8)	1.6 (0.8)
Discussion/interaction	2 (1)	1.8 (0.9)
Practical relevance	2 (1)	1.7 (1)
Personal goal achievement	1.9 (0.9)	1.7 (0.8)
Planning and organization		
Program announcement	1.9 (1.1)	1.5 (0.7)
Selection of dates	2.2 (1.1)	1.7 (0.8)
Time frame	2.1 (1)	2.1 (1.1)
Web conferencing system		
Technical functionality	1.8 (0.7)	—
User friendliness of the screen	1.9 (0.9)	—
Sound quality	1.8 (0.9)	—
Image quality	1.8 (0.8)	—

a Not applicable.

The most named positive aspects of the knowledge modules were the content (n=24), with comprehensive scripts, refreshment of knowledge, and new impulses in a comprehensible manner, and the possibility to have access before and after the materials. The second most named was the good support (n=24), which was fast, friendly, and competent, gave individual help, and was very helpful with further questions. The practical relevance and application orientation were named as a positive aspect, the third most (n=14). In turn, most participants said that they could not name any negative aspects (n=25). Some mentioned that it was too much input for the short period of time (n=7), and others said that the modules had too much frontal teaching (n=6), which made the courses dry, difficult to follow, boring, and with too little interaction.

For the practical modules, the exchange with other participants in small groups was particularly positively highlighted by the Digi-Managers (n=26) for offering new perspectives or solutions in conversations. Furthermore, positively perceived was the very informative and instructive nature of the practice modules (n=10) and direct transfer to everyday practice (n=9). When asked about negative aspects, the most common response was that the participants could not name any (n=12). Some participants said that they wished for better day and time selection options (n=4), because the option selections were confusing, could not always be set up, and the always-changing slots were problematic. Some participants wished for more feedback on the tasks they had completed (n=4).

### Evaluation of the Digital Maturity Tool

The usability of the digital maturity tool was ranked via SUS score with a mean value of 85 (SD 12.9), which could be classified as an A+ grade after the Sauro-Lewis grading scale [28]. The content of the tool was evaluated very positively in the different aspects with values between 1.39 and 1.79 (Table 3).

**Table 3.** Evaluation of the digital maturity tool content aspects by the Digi-Managers (scale from 1 (very good) to 6 (very bad)).

Content evaluation categories	Content rating, mean (SD)
Topicality of content	1.4 (0.6)
Content structure	1.6 (0.7)
Practical relevance	1.6 (0.7)
Personal goal achievement	1.8 (1)

Regarding the digital maturity tool, the participants especially liked the visual representation as a radar chart (n=23) because it was appealing, clear, and colorful; showed all relevant information at a glance; and made the digitalization tangible. A lot of participants positively highlighted the possibility to determine the “status quo” of their practice (n=15) and the potential to uncover gaps and deficits (n=11). Most had no negative aspects (n=16), but some mentioned that the answer options to the items were not always clearly distinguishable, or there were no answer options that fitted their practice perfectly, but it was possible to clarify answers through free-text fields (n=8). Some participants had problems with lots of technical jargon (n=5).

### Progression of Digital Literacy

**Figure 2.**
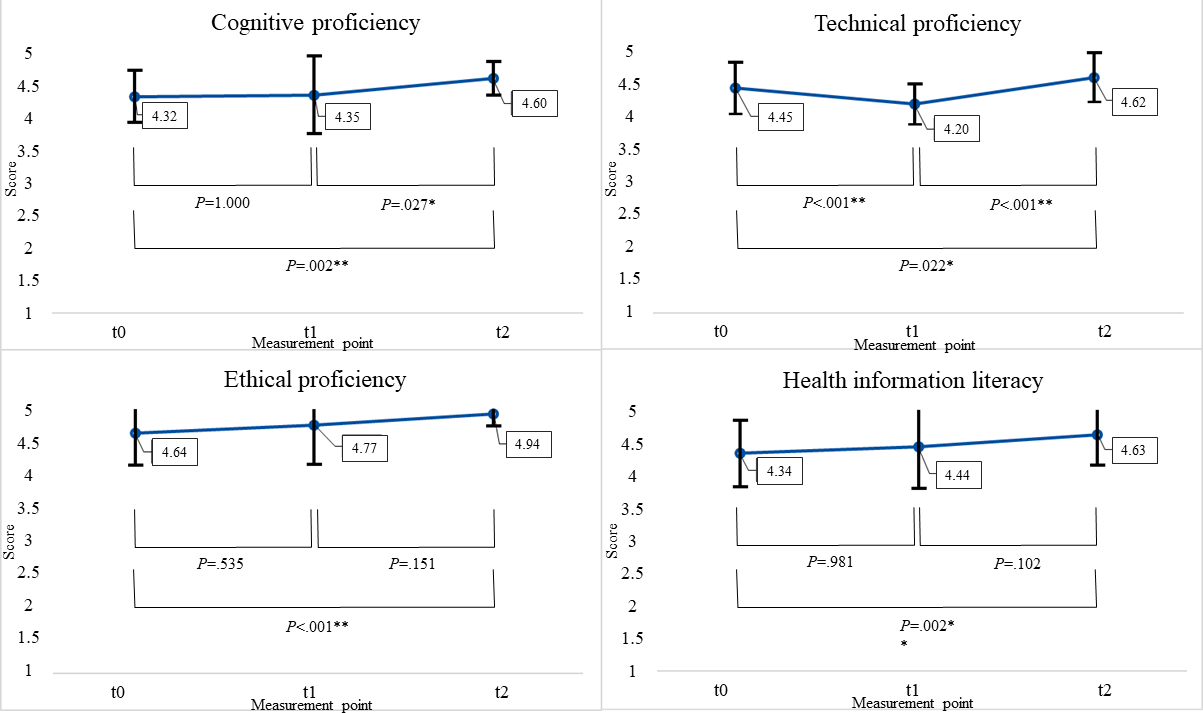
Means, SDs, and post hoc results for the 4 PHIC4PHC (Public Health Informatics Competencies for Primary Health Care) domains. *P<.05, **P<.01.

An ANOVA with repeated measures showed that *cognitive proficiency* changed significantly (*F*_1.67,63.55_=5.5, *P*<.009; partial η²=0.13). ω²=0.1 and ε²=0.1 could be classified as a medium effect. Because of violations of sphericity (*P*=.018), the Greenhouse-Geisser adjustment was used. Bonferroni-adjusted post hoc analysis revealed a significant increase (*P*=.027) in cognitive proficiency from the second to last measurement point (mean_Diff_ –0.25, 95% CI –0.48 to –0.22) and a significant increase (*P*=.002) between the first and last survey (mean_Diff_ –0.28, 95% CI –0.46 to –0.09) (mean_t0_ 4.3, SD_t0_ 0.4; mean_t1_ 4.4, SD_t1_ 0.6; mean_t2_ 4.6, SD_t2_ 0.3) (Figure 2).

*Technical proficiency* initially decreased before rising again at the last measurement point (mean_t0_ 4.5, SD_t0_ 0.4; mean_t1_ 4.2, SD_t1_ 0.3; mean_t2_ 4.6, SD_t2_ 0.4). None of the variables were normally distributed. Because of violations of sphericity, the Greenhouse-Geisser adjustment was used to correct the repeated measures ANOVA, and it showed significant differences (*F*_1.83,75.05_=31.7, *P*<.001; partial η²=0.44). ω²=0.42 and ε²=0.42 showed a large effect. Bonferroni-adjusted post hoc analysis revealed significant differences between all measurement points: a significant decrease (*P*<.001) between the first and second survey (mean_Diff_ 0.25, 95% CI 0.14 to 0.36), as well as a significant increase (*P*<.001) between the second and third survey (mean_Diff_ –0.41, 95% CI –0.54 to –0.28), and an overall significant increase (*P*=.022) between the first and third survey (mean_Diff_ –0.17, 95% CI –0.31 to –0.02).

*Ethical proficiency* increased over the entire training period (mean_t0_ 4.6, SD_t0_ 0.5; mean_t1_ 4.8, SD_t1_ 0.6; mean_t2_ 4.9, SD_t2_ 0.2), with a statistically significant difference between measurements, assessed by repeated measures ANOVA with the Greenhouse-Geisser correction (Mauchly test *P*=.003; *F*_1.68,94.3_=6.54, *P*=.004; partial η²=0.11). ω²=0.09 and ε²=0.09 could be classified as a medium effect. Bonferroni-adjusted post hoc analysis showed a significant increase (*P*<.001) from the first to last measurement point (mean_Diff_ –0.30, 95% CI –0.46 to –0.14).

The change in *health information literacy* was assessed as significant through repeated measures ANOVA (*F*_2,102_=5.71, *P*=.004; partial η²=0.1). ω²=0.08 and ε²=0.08 show a medium effect. Sphericity could be assumed (*P*=.361). Post hoc analysis with the Bonferroni correction revealed a significant increase (*P*=.002) from the first (mean_t0_ 4.3, SD_t0_ 0.5) to last (mean_t2_ 4.6, SD_t2_ 0.5) measurement point (mean_Diff_ –0.29, 95% CI –0.49 to –0.10).

The confidence level for technology usage increased from one measuring time to the next (mean_t0_ 3.6, SD_t0_ 0.8; mean_t1_ 3.7, SD_t1_ 0.6; mean_t2_ 4.1, SD_t2_ 0.6). None of the variables were normally distributed, as assessed by the Shapiro-Wilk test, but since the sample size was sufficiently large (n>30) and due to the robustness of the ANOVA with repeated measures, no further actions need to be taken [29]. The Greenhouse-Geisser adjustment was used to correct the violations of sphericity due to a significant result of the Mauchly test (*P*=.002) [30]. A repeated measures ANOVA with a Greenhouse-Geisser correction determined that the confidence level showed a statistically significant difference between measurements (*F*_1.6,71.72_=22.59, *P*<.001; partial η²=0.33). ω²=0.32 and ε²=0.32 could be classified as a large effect.

Bonferroni-adjusted post hoc analysis revealed significantly (*P*<.001) higher confidence scores in the comparison between the first and last measurement points (mean_Diff_ –0.48, 95% CI –0.32 to 0.03), and significantly (*P*<.001) higher between the second and last measurement points (mean_Diff_ –0.34, 95% CI –0.48 to –0.20).

The attitude toward (health) technology was assessed by TAM and UTAUT items. There was no statistically significant difference for the different measurement points of the TAM (*F*_1.65,70.91_=1.89, *P*=.166) as assessed by repeated measures ANOVA with the Greenhouse-Geisser correction (Mauchly test *P*=.007). The mean TAM score was already high in the first survey (mean_t0_ 6, SD_t0_ 0.7) and remained at a similar height for the second (mean_t1_ 6.2, SD_t1_ 0.7) and third (mean_t2_ 6.3, SD_t2_ 0.8) surveys.

For the UTAUT scores, normal distribution (*P*_t0_=.174; *P*_t1_=.200; *P*_t2_=.200) and sphericity (*P*=.080) could be assumed. The repeated measures ANOVA showed no significant differences (*F*_1.65,70.91_=1.89, *P*=.166). The UTAUT score remained—similar to the TAM score—high over the entire data collection period (mean_t0_ 6, SD_t0_ 0.3; mean_t1_ 6.1, SD_t1_ 0.7; mean_t2_ 6.2, SD_t2_ 0.4).

## Discussion

### Principal Results

The study results showed that the training was both successful and satisfactory for the participants of the certificate course “Certification of Digitalization Officers in Medical Practices and Psychotherapeutic Practices (Digi-Manager).” Although the Digi-Managers already started with a high level of self-evaluated digital literacy, this increased significantly from the beginning to the end of the training program. Hypothesis H1 was confirmed: after the training course, the Digi-Manager had significantly higher values in the cognitive proficiency, ethical proficiency, and health information literacy with a medium effect. The significant increase in technical proficiency even had a large effect. Hypothesis H2 was also confirmed, and attending the Digi-Manager training course significantly improved participants’ general confidence level for technology usage with a large effect. The attitude toward digital (health) applications remained stable at a high positive level over the entire course duration. Therefore, hypothesis H3 was rejected because the attitude toward digital (health) applications did not improve during and after the Digi-Manager training course. We assumed that this was because the practices had to actively apply for the course. Although the participants were finally drawn by lot, it is likely that practices that were interested in digital topics anyway applied more often and sent their most digitally savvy colleague. The very positive perception of the training—both for the knowledge and practical modules—also remained stable throughout the course. Age had no effect on most of the variables. The only variable that was negatively influenced by older age was self-confidence in use. This effect was stable for all 3 measurement times. Similar results of lower self-confidence when dealing with digital topics in older adults were found in other studies [20]. Our survey therefore reflects the state of research that there are hardly any age effects in the use of digital tools. Gender effects could not be examined because of the vast majority of female-identifying participants. However, the low participation rate of men reflects the real gender proportion of men in this occupational field, which was only 2% in the year 2023 in Germany, as reported by the federal employment agency (Bundesagentur für Arbeit).

To our knowledge, this is the first training program for medical assistants for general digital literacy that was scientifically evaluated. Multiple review papers show that training courses for health care staff teach mostly about specific technologies such as electronic medical records or telehealth [31,32], take place for the most part in an academic context [33], or are intended for physicians [34]. Many authors criticize the lack of sufficient training [34-36].

A competence measure study in the more digital-savvy country of Finland [37] showed that further education for health professionals is needed not only in Germany but all over the world. The level of digital competence among health care professionals also varies in other countries. Especially, human-centered remote counseling competence was identified as the category with the weakest score. Health care professionals’ knowledge of ethical, legal, and regulatory requirements, as well as privacy and security issues regarding digital tools, was named as a mandatory subject matter in training. In the study of Jarva et al [37], higher age was associated with lower evaluation of digital solutions as part of work and a decrease in self-evaluated competence. This was not further confirmed in our study.

### Limitations

One limitation of the results is that, despite repeated reminders both digitally and in person, there was a high level of nonparticipation at the time of the last survey. Only 64% (64/100) completed the last survey. It cannot be ruled out that this might slightly distort the results as it is more likely that the committed participants, who got a lot out of the training anyway, participated until the end than those who perceived the training as less enriching. In future training courses, an attempt will be made to combine the evaluation with the last content-related work in the course in order to increase participation in the final evaluation.

As a further limitation on the part of the participants, it should be considered that, as already briefly mentioned above in the discussion of H3, a self-selection bias could exist through the application process and registration through the practice owner. Participants were either self-motivated to take part or were perceived by their practice owner as the “most suitable” and therefore probably the most interested person of the practice in digital topics.

Furthermore, it must be questioned whether the self-assessment questionnaires were really suitable for measuring competence. According to the results, competence in the domain of technical proficiency decreased when participating in this training course, which appears illogical. Since the questionnaire measures how capable the participants see themselves, this value can decrease if they learn what they do not yet know. Self-perception does not always match actual performance [38]. This is further backed up by another study that questions the suitability of self-assessment scales for measuring competence: “Perceived skills [...] do not predict actual performance,” as van der Vaart et al [39] stated in their paper, comparing the results of self-assessment scales that aim to measure the eHealth literacy of participants, with their actual performance in different skill tests [39]. In this study, correlations between the used eHEALS (eHealth Literacy Scale) measure and successfully completed tasks were nonsignificant and weak, and no group differences between participants who scored below and above the median in the performance tests for the eHEALS scores were found. Jarva et al [37] supported this thesis by using the specified term “self-evaluated competence” in their study. The reliability and validity of estimating one’s own competence have already been questioned by many authors [40-42]. Despite this, self-assessment of specific skills and knowledge is, to date, the most commonly used form of measuring digital competence [21]. The question arises as to whether the questionnaires—tested for quality characteristics such as reliability—are in fact simply measuring a different concept than competence. Ulfert-Blank and Schmidt [43] suggested *digital self-efficacy* as a possible characteristic that could be measured through these instruments, defined as “an individual’s perception of efficacy in performing tasks related to the use of digital system.” Bancroft et al [13] proposed that self-assessment of competence measures a mixture of competence and confidence and that both concepts are closely related and sometimes conflated, but also could be out of alignment, and a lack of confidence could hold back people who are per se competent. New paths must be found to measure the actual digital competence of health care professionals.

### Future Research Directions

As mentioned above, new alternative ways to measure digital literacy/competence—besides self-assessment scales—must be found. One promising approach could be the use of performance measures, which were already used for the measurement of other concepts, like eHealth literacy [39] or data literacy [44,45].

It is noticeable among the participants of the training course that they all started with a very positive attitude. This is probably because there was an active application process for the training, and people who were already digitally interested were more likely to want to be trained as Digi-Managers. It would be interesting to see whether further training would lead to an improvement in the attitudes of people who are not yet so positively disposed.

### Learnings for Future Courses

The success in positive learning outcomes and satisfaction of the participants shows the relevance of the continuation of the course. The training program will be carried on with slight changes, following the feedback of participants, instructors, and organizers. Future courses will be shorter in time to enable smaller practices to participate with fewer lost hours of their employees and more e-learning and web-based courses in order to travel less. That should also improve the day and time selection options. Course sizes are to be reduced to enable more active involvement of participants and, above all, to further support practical networking and the exchange of experience. A streamlined concept with consistent quality should focus on the unique selling points of the Digi-Manager training: the digital laboratory and maturity model. The additional focus on soft skills (project management, communication, and conflict management) is helpful for the effective transfer and realization of digital projects in practice.

### Conclusions

The Digi-Manager program was a successful and long-needed training program for health care professionals in the German region of Westfalen-Lippe. More training programs and courses for health care professionals are needed not only in Germany but all over the world. The mixture of transferring theoretical knowledge and practical applications with reference to one’s own everyday work through soft skill training, the maturity model, and digitalization strategy results in a unique and effective further education concept.

## Supplementary material

10.2196/70843 Multimedia Appendix 1 Equivalent of the ÄKWL certificate course and content of the different modules. ÄKWL: Ärztekammer Westfalen-Lippe (medical association of the Westphalia-Lippe region).

10.2196/70843 Multimedia Appendix 2 Questionnaires of the web-based surveys.

10.2196/70843 Multimedia Appendix 3 Inductively formed categories from free-text answers regarding the course modules.

10.2196/70843 Multimedia Appendix 4 Correlation coefficients and significance values.

10.2196/70843 Checklist 1 Checklist items of GREET (Guideline for Reporting Evidence-Based Practice Educational Interventions and Teaching).

## Abbreviations

ÄKWL: Ärztekammer Westfalen-Lippe (medical association of the Westphalia-Lippe region)
eHEALS: eHealth Literacy Scale
GREET: Guideline for Reporting Evidence-Based Practice Educational Interventions and Teaching
KVWL: Kassenärztliche Vereinigung Westfalen-Lippe (association of statutory health insurance physicians of the Westphalia-Lippe region)
mHealth: mobile health
PHIC4PHC: Public Health Informatics Competencies for Primary Health Care
SUS: system usability scale
TAM: technology acceptance model
UTAUT: unified theory of acceptance and use of technology
