# Supplementary material for: Digital Literacy Training for Digitalization Officers (“Digi-Managers”) in Outpatient Medical and Psychotherapeutic Care: Conceptualization and Longitudinal Evaluation of a Certificate Course
Source: JMIR Med Educ. 2025 Aug 29;11:e70843. doi: 10.2196/70843 (PMC12396773; doi:10.2196/70843)
Supplement: Multimedia Appendix 1 [file mededu-v11-e70843-s001.pdf]

Equivalent of the ÄKWL certificate course and content of the different modules

**Module number**	**Module name**	**Equivalent from the certified training course of ÄKWL**[1]	**Module content**
Knowledge Modules			
1	Data protection and data security	Module 1: Data protection and data security	• data protection aspects of the digitalization of medical practices<br>• skills in a digital medical practice - technical, data, information and data protection skills<br>• practical tips for IT security & data protection in the digital world<br>• IT security requirements for medical practices
2	Information and communication technology	Module 2: Information and communication technology	• introduction of IT equipment in medical practices - from EDP to modern IT<br>• exchange of information and data in the healthcare sector
3	Telematic infrastructure (TI) and TI applications	Module 3: Telemedical basics<br>Module 4: Telemedical applications	• introduction to TI (Background, legal framework, overview of mandatory application)<br>• TI applications in detail (eRezept, ePA, KIM, EAU, eArztbrief)<br>• access to TI (TI connector, SMC-B card, EHBA, signature procedure)<br>• TI applications in practice experience<br>• outlook TI (TI 2.0, mobile access to TI, digital identities)<br>• future TI applications (TIM, eBTM prescription)<br>• structured data in the healthcare sector (MIO)
4	Digital strategies and digital maturity model	Module 4: Telemedical applications + add-on	• introduction to digital medicine (telemedicine, DiGA, tele monitoring)<br>• benefits of digital tools for patient care<br>• What are strategies and why do you need them?<br>• “target image” of the KVWL (best practice from the KVWL region)<br>• project, change and conflict management<br>• introduction of maturity level measurement and instruments for

			assessing the degree of digitalization<br>• measuring the degree of digitalization of (medical) practices - KVWL maturity model (digital tool)
Practical Modules			
5	Kick off event	Add-on	• procedure of the practical modules and final thesis
6	Fundamental knowledge	Add-on	• introduction to project management<br>• practice management<br>• IT equipment
7	Topic-specific dipraxis tour	Add-on	• TI<br>• TI security and data protection
8	Individual dipraxis tour/consultation	Add-on	• digital and automated work processes/testing digital tools<br>• digitization potential in the individual practice structure

[1]The ÄKWL training course "Electronic practice communication and telematics" ("Elektronische Praxiskommunikation und Telematik") is a certified further training course for medical assistants in the form of in-service theoretical and practical training with 80 teaching units according to the curriculum of the German Medical Association. Modules 1 and 2 of the model training curriculum are congruent with Module 6 of the advanced training course "Specialist in outpatient medical care" and can therefore be transferred by the state medical associations. Modul 2, 3 and 4 are also creditable for the EVA ("Entlastende Versorgungsassistenz", Relieving care assistance) or NäPa ("Nichtärztliche Praxisassistentin", Non-physician practice assistant) training course. Module 1 is a qualification as data protection officer for outpatient practices.

References

Bundesärztekammer (2024). Musterfortbildungscurriculum für Medizinische Fachangestellte „Elektronische Praxiskommunikation und Telematik". Bundesaeztekammer. https://www.bundesaerztekammer.de/fileadmin/user_upload/_old-files/downloads/pdf-Ordner/MFA/Curr_Telematik.pdf (retrieved 2024, December 19)

Akademie für medizinische Fortbildung der Ärztekammer Westfalen-Lippe und der Kassenärztlichen Vereinigung Westfalen-Lippe (2024, June). Fortbildungen für Medizinische Fachangestellte und Angehörige anderer Medizinischer Fachberufe. Akademie-WL. https://www.akademie-wl.de/fileadmin/MFA_Brosch%C3%BCre_2024_Stand_06.2024.pdf (retrieved 2024, December 19)
