# Supplementary material for: Digital Literacy Training for Digitalization Officers (“Digi-Managers”) in Outpatient Medical and Psychotherapeutic Care: Conceptualization and Longitudinal Evaluation of a Certificate Course
Source: JMIR Med Educ. 2025 Aug 29;11:e70843. doi: 10.2196/70843 (PMC12396773; doi:10.2196/70843)
Supplement: Multimedia Appendix 2 [file mededu-v11-e70843-s002.pdf]

Online Survey (all questionnaires for the three different measurement points)

„Evaluation of a certificate course for the training of digitalization officers in medical and psychotherapeutic practices via a longitudinal study“

Note: The questionnaires were translated into German for the study and translated back into English here for readability.

Participant Characteristics

How old are you?

	Age: ________ years
	Prefer not to say

Which gender do you identify as?

	male
	female
	nonbinary
	no gender
	Prefer not to say

In which specialist area does the practice you are employed by work?

	Specialist area: ____
	Prefer not to say

How long do you work in your field of occupation? Please round up to whole years.

	Work experience: ________ years
	Prefer not to say

Identification code

In order to be able to assign individual parts of the evaluation to each other without violating your anonymity, we ask you to create your individual identification code. This six-digit code is made up of:

- The two day digits of your own birthday,
- the first two letters of your mother's first name,
- the first two letters of your father's first name.

(Example: For a birthday on January 01, 1980 and the parents Paula and Sven, the code is 01PASV)

Identification code: __ __ __ __ __ __

Digital literacy

Please indicate to what extent you agree with the following statements. Please note that some statements are negated (e.g. I do **not** know ...; I **cannot**...).
Rate the statements on the following scale: 1 = do not agree at all, 2 = rather disagree, 3 = neither, 4 = rather agree, 5 = completely agree.

The term digital health systems refers to all systems that are used in the context of a digital practice. These range from practice management systems to desktop applications (e.g. e-mail programs, SharePoint,...).

	1	2	3	4	5
1. I know about different digital health systems.					
2. I know about the digital health systems in my workplace.					
3. I know the milestone in the evolution of digital health systems in my workplace.					
4. I know about the computer applications that can help me to perform the daily tasks.					
5. Computers can be used as a tool for working and controlling.					
6. I know the importance and the advantages of data to my work.					
7. I am concerned about how data have been collected and used.					
8. A digital health system is a tool for health service efficiency in my workplace.					
9. I can use digital health systems to complete my work.					
10. I can use digital health systems to save, retrieve, and transfer data in the workplace					
11. I cannot use computer appliances used in health service and medical service.					
12. I can solve common and simple computer errors.					
13. I do not know how to use the computer for personal use.					
14. I cannot use computerized devices, such as printer or scanner.					
15. I do not know the reason for a slow response of a computer program, such as many users at the same time.					
16. Computer programs are tools for effectiveness and efficiency.					
17. Files on a computer must be backed up.					
18. I know the basic usage of a computer, such as shutting down and using a mouse.					
19. I know how to use an operating system, such as Windows.					
20. I know how to manage and save files.					
21. I do not know how to install drivers for the computer devices, such as printers and scanners.					
22. I know the file management function of operating systems, such as create, copy, move folder or file.					
23. I can use word processing software to process documents for daily tasks.					
24. I cannot use a spreadsheet program (e.g., Excel) to perform simple data analysis.					
25. I cannot use presentation editing software (Power Point) for presentations and education.					
26. I cannot use statistics software for research and daily tasks.					
27. I can use database software to create a database that is needed in my daily task.					
28. I can use a computer as a self-learning tool.					
29. I cannot use computerized self-learning, such as e-learning or CD learning.					
30. I can convert a file to different application formats, such as Word to PDF.					

31. I cannot use the World Wide Web (www) to search information.					
32. I can receive and send emails to transfer files through the network.					
33. I know the importance of confidentiality when processing data in the medical records and on computers.					
34. I know the regulations concerning the protection of patient identity on computers.					
35. I know what kind of health information can be found on the internet.					
36. I know where to find useful health information on the internet.					
37. I know that the internet can be used as the health information resources.					
38. I know how to find useful health information on the internet.					
39. I know how to use the internet to answer questions about health.					
40. I know how to use health information that had been found to help my daily tasks.					
41. I can evaluate health information found on the internet.					
42. I can differentiate between correct and incorrect information found on the internet.					

Indicate how self-confident you feel when using the following items. Rate on the following scale: 1 = not at all confident, 2 = a little confident, 3 = rather confident, 4 = quite confident, 5 = absolutely confident.

	1	2	3	4	5
1. Computer					
2. Microsoft office applications					
3. Smartphones					
4. Tablets					
5. E-Mail					
6. Internet					
7. Social media (z.B. Facebook, Twitter, Instagram,…)					

Please indicate the extent to which you agree with the following statements. Rate the statements on the following scale: 1 = strongly disagree, 2 = disagree, 3 = somewhat disagree, 4 = neither, 5 = somewhat agree, 6 = agree, 7 = strongly agree.

The term digital (health) systems refers to all systems that are used in the context of a digital practice. These range from practice management systems to desktop applications (e.g. e-mail programs, SharePoint,...).

	1	2	3	4	5	6	7
1. Using digital systems would enable me to accomplish tasks more quickly.							
2. Using digital systems in my job would improve my job performance							
3. Using digital systems in my job would increase my productivity.							
4. Using digital systems would enhance my effectiveness on the job.							
5. Using digital systems would make it easier to do my job.							
6. I would find digital systems useful in my job.							
7. Learning to operate digital systems would be easy for me.							
8. I would find it easy to get information systems to do what I want them to do.							
9. My interaction with digital systems would be clear and understandable.							

10. I would find digital systems to be flexible to interact with.							
11. It would be easy for me to become skillful at digital systems.							
12. I would find digital systems easy to use.							

Please indicate the extent to which you agree with the following statements. Rate the statements on the following scale: 1 = strongly disagree, 2 = disagree, 3 = somewhat disagree, 4 = neither, 5 = somewhat agree, 6 = agree, 7 = strongly agree.

The term digital (health) systems refers to all systems that are used in the context of a digital practice. These range from practice management systems to desktop applications (e.g. e-mail programs, SharePoint,...).

	1	2	3	4	5	6	7
1. Using digital systems is a good idea.							
2. Working with digital systems is fun.							
3. I like working with digital systems.							
4. People who influence my behaviour think that I should use digital systems.							
5. People who are important to me think that I should use digital systems.							
6. The senior management of the organisation have been helpful in the use of digital systems.							
7. In general, the organisation has supported the use of digital systems.							
8. I have the necessary resources to use digital systems.							
9. I have the knowledge necessary to use digital systems.							
10. Digital systems are not compatible with other systems I use.							
11. A specific person (or group) is available for assistance with digital systems difficulties.							
12. I feel apprehensive about using digital systems.							
13. It scares me to think that I could lose a lot of information using digital systems by hitting the wrong key.							
14. I hesitate to use digital systems for fear of making mistakes I cannot correct.							
15. Digital systems are somewhat intimidating to me.							

Evaluation of knowledge modules

Please rate the following points on the following scale: 1 = very good, 2 = good, 3 = rather good, 4 = rather not good, 5 = bad, 6 = very bad.

		1	2	3	4	5	6
**planning and organization**	program announcement						
	selection of dates						
	time frame						
**support and design**	participant support						
	technical moderation						
	quality of scripts						
	atmosphere						
**web conferencing system**	technical functionality						
	user-friendliness of the screen						
	sound quality						
	image quality						
**content**	topicality of content						
	content structure						
	selection of speakers/authors						
	discussion/interaction						
	practical relevance						
	personal goal achievement						

Here you have the opportunity to report in your own words what you particularly liked about the knowledge modules:

____

____

____

____

____

Here you have the opportunity to report in your own words what you did not like so much about the knowledge modules:

____

____

____

____

____

Evaluation of practice modules

Please rate the following points on the following scale: 1 = very good, 2 = good, 3 = rather good, 4 = rather not good, 5 = bad, 6 = very bad.

		1	2	3	4	5	6
**planning and organization**	program announcement						
	selection of dates						
	time frame						
**support and design**	participant support						
	atmosphere						
**content**	topicality of content						
	content structure						
	selection of speakers/authors						
	discussion/interaction						
	practical relevance						
	personal goal achievement						

Here you have the opportunity to report in your own words what you particularly liked about the practice modules:

Here you have the opportunity to report in your own words what you did not like so much about the practice modules:

Evaluation of the maturity model

Please indicate the extent to which you agree with the following statements. Rate the statements on the following scale: 1 = strongly disagree, 2 = somewhat disagree, 3 = neither, 4 = somewhat agree, 5 = strongly agree.

	1	2	3	4	5
1. I think that I would like to use this system frequently.					
2. I think that I would like to use this system frequently.					
3. I thought the system was easy to use.					
4. I think that I would need the support of a technical person to be able to use this system.					
5. I found the various functions in this system were well integrated.					
6. I thought there was too much inconsistency in this system.					
7. I would imagine that most people would learn to use this system very quickly.					

8. I found the system very awkward to use.					
9. I felt very confident using the system.					
10. I needed to learn a lot of things before I could get going with this system.					

Please rate the following points on the following scale: 1 = very good, 2 = good, 3 = rather good, 4 = rather not good, 5 = bad, 6 = very bad.

		1	2	3	4	5	6
**content**	topicality of content						
	content structure						
	selection of speakers/authors						
	discussion/interaction						
	practical relevance						
	personal goal achievement						

Here you have the opportunity to report in your own words what you particularly liked about the maturity model:

____

____

____

____

____

Here you have the opportunity to report in your own words what you did not like so much about the maturity model:

____

____

____

____

____
