# Supplementary material for: Digital Literacy Training for Digitalization Officers (“Digi-Managers”) in Outpatient Medical and Psychotherapeutic Care: Conceptualization and Longitudinal Evaluation of a Certificate Course
Source: JMIR Med Educ. 2025 Aug 29;11:e70843. doi: 10.2196/70843 (PMC12396773; doi:10.2196/70843)
Supplement: Multimedia Appendix 3 [file mededu-v11-e70843-s003.pdf]

Inductively formed categories from free text answers regarding the course modules

S1. Particularly positive aspects of the knowledge modules

Category	Description	Number of nomination
contents	comprehensive scripts, great working material, new impulses/suggestions or refreshing of knowledge, access of scripts for re-reading, not dry, comprehensible regardless of prior knowledge, change of perspective	24
support	helpfulness with questions, individual help, fast, friendly, competent	17
application orientation	practical relevance	14
virtual events	nevertheless (or precisely because of this) great discussions possible, chat function, journey would be far	11
lecturers	enthusiasm, exciting teaching, understandable explanations, sympathetic, telling from own experience, friendly, helpful, questions were answered quickly and comprehensively	10
exchange with other participants	collaboration, working in groups, lively discussions	7
topicality		6
self-scheduling	flexible choice of dates	5
insights into future prospects	impetus for further digitization plans	2
face-to-face events	alternative locations offered	2
atmosphere		2
structure and organization		1
self-study part		1
hybrid event	both eLearning and practice	1
all/ overall satisfied		8
nothing		1

S2. Particularly negative aspects of the knowledge modules

Category	Description	Number of nomination
nothing		25
Too much scope/input	In relation to period of time for the course	7
Too much frontal teaching	dry, difficult to understand, boring lectures, more interaction desired	6
Difficulties with eLearning	2x internet connection, 2x sound, 1x unspecified	5
Spontaneous changes to times/work assignments		4
Too much online	More face-to-face events desired	4
Homework before the course (module 4)	More instruction/feedback needed, prior presence recommended	4
Long units	difficulty concentrating, more breaks needed	3
Module 4 too theoretical	Content very unfamiliar and therefore difficult as a self-study phase, more practical relevance	3
Transparency of the next work steps	Unclear planning: Which tasks are still open, which appointments still need to be booked?	3
Availability of days and times		3
Repetitions	e.g. for module 1	2

Module 2 not particularly instructive	Criticism of lecturer and topicality of teaching materials	2
No possibility to work ahead	Scripts should be made available before the course	2
Problems with appointment booking	Unintentional appointment booking,<br>Few free dates as a successor	2
Problems in the process		1
Announce times and dates earlier		1
Theory and everyday practice not compatible		1
More precise topics	"Less beating about the bush"	1
Material not always available afterwards		1
Group work with strangers online	Took a lot of time	1
Discussions too long	Partial lack of qualification of the lecturers	1
No fixed dates for eLearning	Employer cannot give time off like this	1
eLearning too difficult		1
eLearning as an unfamiliar format		1
Everything desired online		1

S3. Particularly positive aspects of the practice modules

**Category**	**Description**	**Number of nomination**
exchange with other participants	offers new perspectives or solutions open up in conversation, small groups are very pleasant	26
very instructive/informative		10
directly transferable to everyday practice		9
opportunity to ask questions		8
great support	friendliness, professional competence, good explanations	7
practical testing what options/tools are available		3
atmosphere	relaxed and pleasant	2
new aspects/impulses		2
good organization		2
test other medical practice management systems		1
project planning is now fun		1
presence courses		1
relevance of the topics		1
varied topics		1
everything		1

S4. Particularly negative aspects of the practice modules

**Category**	**Description**	**Number of nomination**
nothing		12
Day selection and time	Confusing, cannot always be set up, always changing slots	4

Missing feedback		4
More presence/exchange desired		3
Too much time required	High number of hours, some topics too long	3
Group work	Tough and exhausting, group work could put people off further training	2
Topic IT security too detailed		2
Long journey	Suggestion: Complete daily program to make the trip worthwhile	2
More content for practice management desired		1
Communication about desired scope of e.g. final thesis unclear		1
Only common medical practice management systems considered		1
Desire for Excel course offer		1
Desire for topics such as practice website or Google ratings		1
Too little time for topics		1
Could have been more technical		1
Partly dry		1
A lot of information regarding electronic prescriptions	Too much repetition	1
Too little practical relevance		1
Homework in the run-up was difficult		1
More learning-by-doing		1
More support for own implementation desired		1
Too much in presence, except for dipraxis visit everything could have been digital		1
Modules structured very differently		1

S5. Particularly positive aspects of the maturity level tool

**Category**	**Description**	**Number of nomination**
visual representation as a diagram/graphic	appealing, clear, colorful, relevant information at a glance, visual, makes digitization tangible	23
determine the status quo of the practice	"Where do we stand?"	15
uncover "gaps/deficits"		11
determine prospects/potential for the practice	useful and practicable, tool for planning next measures	10
comprehensible and self-explanatory		6
easy to use		6

structuring, division into categories	different areas considered, knowing “what is possible where”?	6
regular use to make progress visible	future use of the tool is planned (comparison of different points in time)	5
can be customized for practice		2
suitable for everyday use		1
objective evaluation possible		1
motivates further research		1
predefined questions		1
everything		1

S6. Particularly negative aspects of the maturity level tool

**Category**	**Description**	**Number of nomination**
no negative aspects		16
answer options not always accurate / clearly defined	must then be supplemented or adapted in the free text, levels poorly delimited from each other by many sub-items	8
lots of technical jargon	sometimes too technical	5
not geared towards specialist practices	some questions not applicable (e.g. video consultation not feasible in some specialist practices)	4
tool crashed and did not save		3
sentences/examples complex to understand		2
desired function: Be able to save and compare different progress statuses		2
color highlighting of the individual points would be nice		2
text fields too small/cluttered		2
questions can only be answered with help	especially technical/data protection	1
lack of individual customizability		1
not all digital areas considered	e.g. X-ray, digital appointment scheduler, digital waiting room, quarterly billing	1
it seemed complicated at first		1
gaps in knowledge were emphasized	“I don't feel like a digital manager”	1
free text answers		1
financial management too specific	topic is rather part of management	1
not designed for cell phone use		1
offer more levels (more selective)		1
function request: Clicking on diagram leads to question category		1
model contains gaps		1
nothing		1
