# Supplementary material for: Digital Literacy Training for Digitalization Officers (“Digi-Managers”) in Outpatient Medical and Psychotherapeutic Care: Conceptualization and Longitudinal Evaluation of a Certificate Course
Source: JMIR Med Educ. 2025 Aug 29;11:e70843. doi: 10.2196/70843 (PMC12396773; doi:10.2196/70843)
Supplement: Multimedia Appendix 4 [file mededu-v11-e70843-s004.pdf]

Correlation coefficients and significance values, * < .05, ** p <.01

	Age	Cogn. prof_S1	Cogn. prof_S2	Cogn. prof_S3	Techn. prof_S1	Techn. prof_S2	Techn. prof_S3	Ethical prof_S1	Ethical prof_S2	Ethical prof_S3	HI lit_S1	HI lit_S2	HI lit_S3	TAM_S 1	TAM_S 2	TAM_S 3	UTAUT _S1	UTAUT _S2	UTAUT _S3	Confid._ S1	Confid._ S2	Confid._ S3
Age	1																					
Cogn. prof_S1	.167	1																				
Cogn. prof_S2	.019	.263*	1																			
Cogn. prof_S3	.117	.159	.303*	1																		
Techn. prof_S1	.070	.442**	.158	.221	1																	
Techn. prof_S2	-.140	.311*	.754**	.185	.351**	1																
Techn. prof_S3	.099	.275	.173	.206	.538**	.522**	1															
Eth. prof_S1	-.106	.180	.114	.096	.133	.147	-.097	1														
Eth. prof_S2	-.110	.027	.698**	.168	-.022	.670**	-.041	.212*	1													
Eth. prof_S3	.002	.208	-.003	.473**	.037	-.013	.353*	.216	-.005	1												
HI lit_S1	-.072	.445**	.145	.201	.371**	.168	.242	.374**	.121	.188	1											
HI lit_S2	-.170	.275*	.570**	.158	.151	.663**	.172	.222*	.595**	-.011	.465**	1										
HI lit_S3	-.099	.161	.150	.439**	.074	.177	.188	.045	.068	.413**	.365**	.360**	1									
TAM_S1	-.036	.555**	.250*	.328*	.521**	.327**	.352*	.090	.100	.311*	.483**	.417**	.487**	1								
TAM_S2	-.131	.249*	.331**	.274	.277*	.354**	.243	.002	.177	.128	.368**	.476**	.255	.611**	1							
TAM_S3	-.038	.191	.185	.210	.171	.264	.074	.009	.102	.110	.063	.419**	.361**	.395**	.559**	1						
UTAUT_S1	-.104	.491**	.103	.511*	.390*	.051	.507*	.241	-.004	.544*	.593**	.156	.562**	.598**	.390*	.424	1					
UTAUT_S2	-.127	.353	.242	.122	.288	.271	.541*	.357*	.132	-.163	.553**	.488**	.259	.522**	.754**	.305	.648**	1				
UTAUT_S3	.117	.300	.287	.206	.198	.319	.492**	-.075	.134	.271	.144	.372*	.390*	.336	.329	.405*	.405	.832**	1			
Confid._S1	-.422**	.354**	.162	.099	.557**	.388**	.307*	.094	.057	.156	.347**	.241*	.189	.480**	.512**	.189	.298	.272	-.005	1		
Confid._S2	-.423**	.348**	.223	.071	.388**	.402**	.068	.077	.141	-.004	.307**	.359**	.202	.422**	.568**	.439**	.270	.458*	-.036	.781**	1	
Confid._S3	-.381**	.240	.090	.182	.268	.318	.338*	.028	.027	.407**	.282*	.390*	.355**	.322*	.475**	.494**	.419	.465	.214	.680**	.796**	1
