# Supplementary material for: Digital Literacy Training for Digitalization Officers (“Digi-Managers”) in Outpatient Medical and Psychotherapeutic Care: Conceptualization and Longitudinal Evaluation of a Certificate Course
Source: JMIR Med Educ. 2025 Aug 29;11:e70843. doi: 10.2196/70843 (PMC12396773; doi:10.2196/70843)
Supplement: Checklist 1 [file mededu-v11-e70843-s005.pdf]

Checklist items of GREET (Guideline for Reporting Evidence-based practice Educational interventions and Teaching)

**Category**	**Items**	**Application (in paper)**
BRIEF NAME	1. INTERVENTION: Provide a brief description of the educational intervention for all groups involved [e.g. control and comparator(s)].	Methods - Course Concept "Certification of Digitalization Officers in Medical Practices and Psychotherapeutic Practices (Digi-Manager)"
WHY - this educational process	2. THEORY: Describe the educational theory (ies), concept or approach used in the intervention.	Introduction
	3. LEARNING OBJECTIVES: Describe the learning objectives for all groups involved in the educational intervention.	Methods - Course Concept "Certification of Digitalization Officers in Medical Practices and Psychotherapeutic Practices (Digi-Manager)"
	4. EBP CONTENT: List the foundation steps of EBP (ask, acquire, appraise, apply, assess) included in the educational intervention.	Introduction & Methods - Course Concept "Certification of Digitalization Officers in Medical Practices and Psychotherapeutic Practices (Digi-Manager)"
WHAT	5. MATERIALS: Describe the specific educational materials used in the educational intervention. Include materials provided to the learners and those used in the training of educational intervention providers	Methods - Course Concept "Certification of Digitalization Officers in Medical Practices and Psychotherapeutic Practices (Digi-Manager)"
	6. EDUCATIONAL STRATEGIES: Describe the teaching/learning strategies (e.g. tutorials, lectures, online modules) used in the educational intervention.	Methods - Course Concept "Certification of Digitalization Officers in Medical Practices and Psychotherapeutic Practices (Digi-Manager)"
	7. INCENTIVES: Describe any incentives or reimbursements provided to the learners.	Methods - Course Concept "Certification of Digitalization Officers in Medical Practices and Psychotherapeutic Practices (Digi-Manager)"
WHO PROVIDED	8. INSTRUCTORS: For each instructor(s) involved in the educational intervention describe their professional discipline, teaching experience/expertise. Include any specific training related to the educational intervention provided for the instructor(s).	All knowledge modules were taught by different instructors, each experts for their own module through working in digital health, data protection and security and/or project management. Most instructors had prior teaching experience within the academic field or further education or had business consulting experience for healthcare practices.
HOW	9. DELIVERY: Describe the modes of delivery (e.g. face-to-face, internet or independent study package) of the educational intervention. Include whether the intervention was provided individually or in a group and the ratio of learners to instructors.	Methods - Course Concept "Certification of Digitalization Officers in Medical Practices and Psychotherapeutic Practices (Digi-Manager)"

WHERE	10. ENVIRONMENT: Describe the relevant physical learning spaces (e.g. conference, university lecture theatre, hospital ward, community) where the teaching/learning occurred.	The online educational materials and virtual classes were distributed by the learning management platform ILIAS and the classroom courses took place in conference rooms of KVWL and ÄKWL. The practical modules took place at the *dipraxis,* a digital laboratory for testing digital tools and analyzing processes in outpatient care.
WHEN and HOW MUCH	11. SCHEDULE: Describe the scheduling of the educational intervention including the number of sessions, their frequency, timing and duration.	Figure 1. Schedule of the certificate course
	12. Describe the amount of time learners spent in face to face contact with instructors and any designated time spent in self-directed learning activities.	Figure 1. Schedule of the certificate course & Methods - Course Concept "Certification of Digitalization Officers in Medical Practices and Psychotherapeutic Practices (Digi-Manager)"
PLANNED CHANGES	13. Did the educational intervention require specific adaptation for the learners? If yes, please describe the adaptations made for the learner(s) or group(s).	There was no need to specifically adapt the course to the learners and no modifications during the course were needed.
UNPLANNED CHANGES	14. Was the educational intervention modified during the course of the study? If yes, describe the changes (what, why, when, and how).	The intervention was not modified during the course of the study.
HOW WELL	15. ATTENDANCE: Describe the learner attendance, including how this was assessed and by whom. Describe any strategies that were used to facilitate attendance.	Methods - Course Concept "Certification of Digitalization Officers in Medical Practices and Psychotherapeutic Practices (Digi-Manager)"
	16. Describe any processes used to determine whether the materials (item 5) and the educational strategies (item 6) used in the educational intervention were delivered as originally planned.	Methods - Procedure
	17. Describe the extent to which the number of sessions, their frequency, timing and duration for the educational intervention were delivered as scheduled (item 11).	The number of sessions, their frequency, timing and duration were delivered as scheduled.
